# 3D Mapping of the SPRY2 Domain of Ryanodine Receptor 1 by Single-Particle Cryo-EM

**DOI:** 10.1371/journal.pone.0025813

**Published:** 2011-10-05

**Authors:** Alex Perálvarez-Marín, HanShen Tae, Philip G. Board, Marco G. Casarotto, Angela F. Dulhunty, Montserrat Samsó

**Affiliations:** 1 Department of Anesthesia, Brigham and Women's Hospital, Harvard Medical School, Boston, Massachusetts, United States of America; 2 Centre d'Estudis Biofísics, Universitat Autònoma de Barcelona, Barcelona, Spain; 3 John Curtin School of Medical Research, Australian National University, Canberra, Australia; 4 Department of Physiology and Biophysics, Virginia Commonwealth University, Richmond, Virginia, United States of America; University of Cambridge, United Kingdom

## Abstract

The type 1 skeletal muscle ryanodine receptor (RyR1) is principally responsible for Ca^2+^ release from the sarcoplasmic reticulum and for the subsequent muscle contraction. The RyR1 contains three SPRY domains. SPRY domains are generally known to mediate protein-protein interactions, however the location of the three SPRY domains in the 3D structure of the RyR1 is not known. Combining immunolabeling and single-particle cryo-electron microscopy we have mapped the SPRY2 domain (S1085-V1208) in the 3D structure of RyR1 using three different antibodies against the SPRY2 domain. Two obstacles for the image processing procedure; limited amount of data and signal dilution introduced by the multiple orientations of the antibody bound in the tetrameric RyR1, were overcome by modifying the 3D reconstruction scheme. This approach enabled us to ascertain that the three antibodies bind to the same region, to obtain a 3D reconstruction of RyR1 with the antibody bound, and to map SPRY2 to the periphery of the cytoplasmic domain of RyR1. We report here the first 3D localization of a SPRY2 domain in any known RyR isoform.

## Introduction

RyR1 consists of 4 subunits of 565 KDa associated in a homotetramer (2.26 MDa) with fourfold symmetry. RyR1 acts as a docking station for proteins and small molecules both in the cytosol and the sarcoplasmic reticulum (SR). The available interacting surface in the SR is limited because of the small mass protruding into the SR lumen, however the cytosolic volume available for protein-protein interaction is huge. Examples of proteins interacting with RyR1 in the cytosolic side include calmodulin [1], [2], [3], FKBP12 [4], [5], the dihydropyridine receptor DHPR [1], [6], [7], and RyR1 itself [8], [9]. Protein-protein interaction domains such as MIR, leucine zippers, EF-hands and SPRY motifs are present in RyR1, several of which are repeated along RyR1's five thousand residue sequence [10].

The SPRY domain has been proposed as a targeting module for protein-protein interactions [11], [12], [13]. The SPRY motif was first identified as a repeat in the splA kinase of *Dictyostelium discoideum* and in the RyR sequences [14]. There are eleven distinct protein families known to contain this domain, which participate in diverse physiological functions such as immunity, development, and signal transduction [15], [16]. The generic structure of SPRY consists of a β-sandwich formed by two four-stranded antiparallel β-sheets. The two β-sheets are interconnected by α-helices, whereas the β-strands are connected by unstructured loops and turns [17]. There are three SPRY domains present in the sequence of RyR1: SPRY1 (residues 582–798), SPRY2 (residues 1085–1208), and SPRY3 (residues 1358–1571) with sequence identities ranging from 10 to 30%. Here we set to map the 3D structure of the SPRY2 domain in the 3D structure of RyR1. The SPRY2 domain has been suggested to play a role in the interaction between the RyR1 and the DHPR [11], [18], [19], [20].

Due to RyR1's large size, electron microscopy (EM) has been the most helpful tool for its structure determination [21], [22], [23], [24], [25]. In the present study, we have combined antibody labeling and single particle cryo-EM to map the position of the SPRY2 domain in RyR1. We have used three different specific antibodies against the SPRY2 epitope in order to determine the positioning of this protein-protein interacting module implicated in the interaction between RyR1 and DHPR.

In several instances, antibody mapping and image reconstruction of proteins has been used to identify certain protein regions. Some examples using negative staining are the DHPR, F_1_ ATPase, and scorpion hemocyanin [26], [27], [28]. In another example, a domain within RyR1 was labeled using cryo-EM [29]. Immunodetection and EM have been previously used to map protein regions using standard 2D or 3D reconstruction methods [26], [27], [28], [29], [30]. In the present study we have developed a new signal enhancement method to ease the 3D determination of the antibody-binding site.

## Results

### Assessment of the Antibodies' Immunoreactivity

Throughout the study we have used three different antibodies against the SPRY2 domain. The first one (anti-SPRY2-A) is a polyclonal antibody against the unstructured loop between two of the β-strands for SPRY2 (residues Pro^1107^-Ala^1121^). Anti-SPRY2-B and anti-SPRY2-C are, respectively, a polyclonal and a monoclonal antibody against the whole SPRY2 domain.

**Figure 1:**
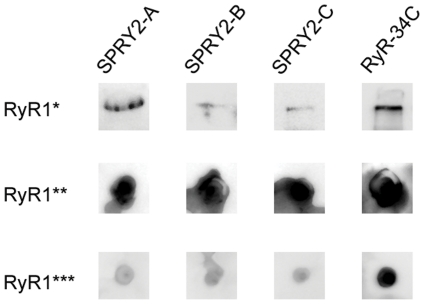
Immunoblotting of RyR1. Immunodetection of the SPRY2 domain in purified RyR1 samples after SDS-PAGE (*) and native conditions (**) and in rabbit muscle vesicles (RyR1 in native conditions) (***). The antibodies used are the following: anti-SPRY2-A, anti-SPRY2-B, anti-SPRY2-C and anti-RyR-34C.

First of all, we qualitatively assessed the ability of the different anti-SPRY2 antibodies to specifically recognize the SPRY2 domain in RyR1. Even though RYR1 contains three structural SPRY domains, the sequence conservation among them is very low, thus unspecific binding is less plausible. Nevertheless for further details on the specificity of anti-SPRY2 antibodies one should refer to [31]. For a cryo-EM study it is important to ensure that the antibodies recognize the SPRY2 epitope in its native conformation, folded within RyR1. Therefore, the SPRY2 domain detection was also carried out in native conditions using dot blot. As a control antibody, we used a commercial antibody against RyR1, antibody 34C. In Western blot, the signal for the detection of the SPRY2 domain in purified, denatured RyR1 was weak as compared to the control RyR1 antibody (34C) (Fig. 1 upper row). In contrast, native dot blot detection of purified RyR1 was very intense in all cases (Fig. 1 middle row), suggesting strong native-structure dependence for the immunodetection. The native dot blot detection levels in rabbit skeletal muscle vesicles were similar amongst all three anti-SPRY2 antibodies, but lower than the 34C antibody (Fig. 1 bottom row). At least for anti-SPRY B, all this is in full agreement with the report that this antibody recognizes native RyR1 in SR vesicles and that it can immunoprecipitate purified RyR1 with a third of the efficacy measured for the antibody 34C [31].

### Cryo-Electron Microscopy

**Figure 2:**
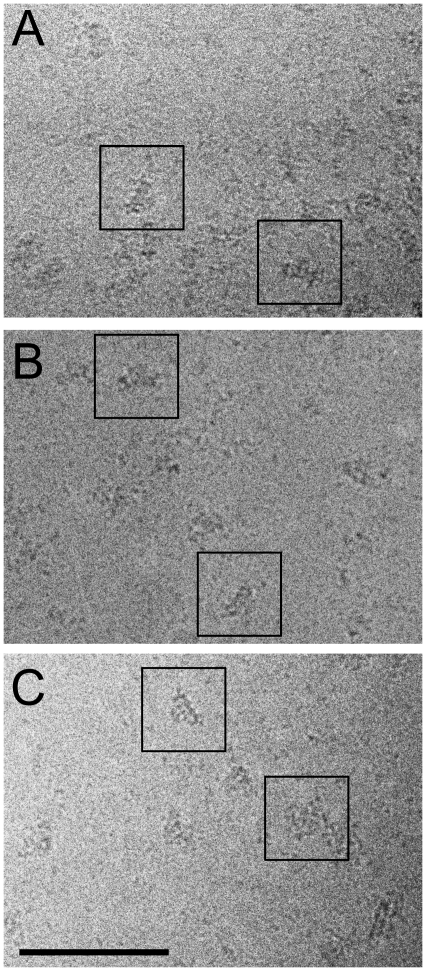
Cryo-EM fields of ice-embedded particles of RyR1 incubated with anti-SPRY2 antibodies. *A.* Anti-SPRY2-A antibody. *B.* Anti-SPRY2-B antibody. *C.* Anti-SPRY2-C antibody. Scale bar, 100 nm.

Once the interaction between folded RyR1 and the different antibodies was assessed, we proceeded to the incubation of purified, solubilized RyR1 with the different antibodies and vitrification of the preparation into a thin ice layer. To increase the randomness of orientations, we applied the sample to holey grids without carbon support. The cryo-preparation yielded well-preserved particles (Fig. 2), and the number of micrographs recorded for each RyR1-antibody preparation were 11, 35 and 49 for anti-SPRY2-A, anti-SPRY2-B and anti-SPRY2-C, respectively.

### Single-Particle Image Processing

**Figure 3:**
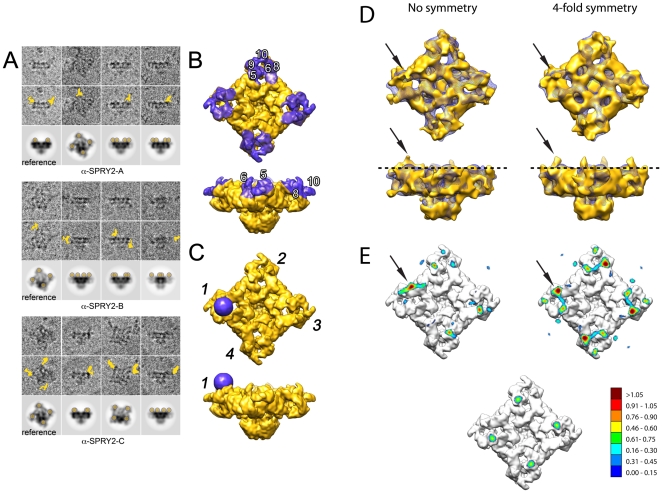
3D reconstruction of the RyR1 anti-SPRY2 binding site. *A.* Unprocessed RyR1 particles incubated with the specified anti-SPRY2 antibody (raw images in upper row, antibody position indicated in yellow in middle row) compared to a calculated 2D projection (bottom row) for RyR1 containing four 30 Å radius spheres at the preliminary proposed binding region for the anti-SPRY2 antibody. For easier visualization the projection of the spheres has been highlighted in semitransparent yellow. *B.* 3D volumes of RyR1 displayed in two different orientations illustrating the preliminary location for the SPRY2 domain (purple shadowing). *C.* Preliminary assigned location for the anti-SPRY2 antibody (purple sphere) in the RyR1 3D reconstruction, at the vicinity of the domains 5 and 6. A set of four such spheres in the first, second, third and fourth repeats of the RyR1 (indicated by numerals) originated the 2D projections shown in the bottom rows of panel A. *D.* Low-resolution 3D reconstructions of RyR1 with anti-SPRY2 antibody (golden surface) in two orthogonal positions, without (left) and with (right) the use of fourfold symmetry. The black arrows point at the main difference between the 3D maps corresponding to the RyR1-antibody and the RyR1 control (purple mesh). *E.* 3D reconstruction with superimposed contour maps indicating the density level in the selected one-pixel slice (dashed line in panel D) for the non symmetrized and the fourfold symmetrized 3D reconstructions. The corresponding contour map for the control 3D volume (panel D, purple mesh) is displayed as a reference (bottom). The gradient scale indicates the density level in arbitrary units.

Individual particles were selected using the program BOXER [32]. If there appeared to be extra mass around the RyR1 particle, presumably due to antibody bound, we placed the center of the window in the center of mass of RyR1 itself. The total number of particles obtained was 189, 490 and 1331 for the RyR1 incubated with anti-SPRY2-A, anti-SPRY2-B and anti-SPRY2-C, respectively. These were subsequently analyzed using the SPIDER/WEB software package [33]. First, a library of 2D projections was constructed by projecting a reference RyR1 [22] (EMDB code 5014) in all possible orientations, at 10-degree intervals, followed by a multi-reference alignment scheme. After visual inspection of every aligned single particle, we identified RyR1s clearly showing extra mass. A representative set of RyR1-antibody particles for anti-SPRY2-A, anti-SPRY2-B and anti-SPRY2-C is shown in Fig. 3A and the full set in Figure S1. These single particles represent RyR1 with at least one antibody attached. In all the three antibody experiments, the antibody binds almost exclusively to the corners of the square-prism shaped cytoplasmic domain, and facing the T-tubule. In the RyR1 domain nomenclature, the three antibodies bind in the vicinity of domains 5, 6, 8, 9 and 10 of RyR1 (Fig. 3B).

### Signal Enhancement of Partially Occupied Symmetry-Related Binding Sites

To further characterize the particles containing bound antibody, we created a series of reference 3D volumes that consisted of the addition of four 30 Å radius spheres at the fourfold symmetrically related potential antibody-binding sites to the reference RyR1 3D reconstruction (see Fig. 3C). Each position of the four-sphere set with respect to the RyR1 was guided from the location of the extra mass taken from the 2D raw images. Given the known flexibility of bound antibodies [34] these occupied a fairly large area and thus only a limited set of reference 3D volumes was necessary to recreate the possible locations. Then, for each raw particle, we calculated the 2D projections of the fabricated RyR1-antibody complex at matching Euler angles (Fig. 3A, Figure S1). These images were then used as a guide to interpret the images of RyR1 with one, two and even three antibodies bound (Fig. 3A). Figure 3C shows the 3D representation that best satisfied all the raw images, with the SPRY2 domain located between domains 5 and 6 of RyR1. Although in this figure the binding site for the antibody is represented by a single sphere at position (repeat) 1, it is worth mentioning that RyR1 is a homotetramer, thus, the antibody binding site is present three more times (repeats 2 to 4 in Fig. 3C).

Besides the number of particles with antibody bound to RyR1 being insufficient to generate a high quality 3D reconstruction, a further limitation was imposed by the fact that while the RyR1 exhibits fourfold symmetry, in general not all its corners were decorated with an antibody. This caused a selective dilution of the signal in the region of the antibody. To overcome this, the symmetry operator and the Euler angles of every single particle-containing antibody were taken into account and re-computed to make the antibody converge onto the same repeat in 3D (repeat 1 in Fig. 3C indicated by a purple sphere). Because there were some RyR1s with two and even three antibodies bound, the dataset expanded from 90 to 124 particles with the SPRY2 antibody in the first repeat. The RyR1-antibody raw images were grouped into a single dataset since the three antibodies bound to the same region of RyR1, and the variance in position within a given antibody dataset was of the same magnitude than the intra-antibody position variance.

With the transformed dataset having all particles with an anti-SPRY antibody bound to repeat 1 of RyR1, we followed two different approaches to obtain a 3D reconstruction, without and with symmetry enforcement. Figure 3D shows both the symmetry-free and the fourfold symmetrized 3D reconstructions of the RyR1 with anti-SPRY2 antibodies bound. The 3D reconstruction without the use of symmetry shows the signal of the antibody concentrated in the first repeat of RyR1, while the fourfold symmetry distributes the signal of the antibody originating from one corner into the four symmetrically-related repeats. In both cases the quality of both reconstructions is poor because of (i) the low number of particles, and (ii) the noise introduced by the flexibility and the diversity of orientations that is adopted by bound antibodies [34]. Nevertheless, a clear signal emerges in the vicinity of domain 6 of RyR1 in both cases. To better illustrate these features, we used a one-pixel thick slice of the 3D reconstruction (dashed line in Fig. 3D) and color-coded the density gradient above a threshold level, thus revealing the more intense regions (Fig. 3E). In the case of the symmetry-free reconstruction, the density is most intense in domain 6 of the first and third repeats but some intensity is also measurable in domains 5 and 10 in the second and fourth repeats (Fig. 3E top left). When fourfold symmetry is applied, the maximum intensity appears in domain 6, followed by domain 5 and 10 with less intensity (Fig. 3E top right). As a control, the same slice is represented for the reference 3D volume (without antibody) showing that the maximum intensity is restricted to domain 5 (Fig. 3E, bottom).

### Docking of the Atomic Structure of the SPRY Domain within RyR1's 3D Envelope

**Figure 4:**
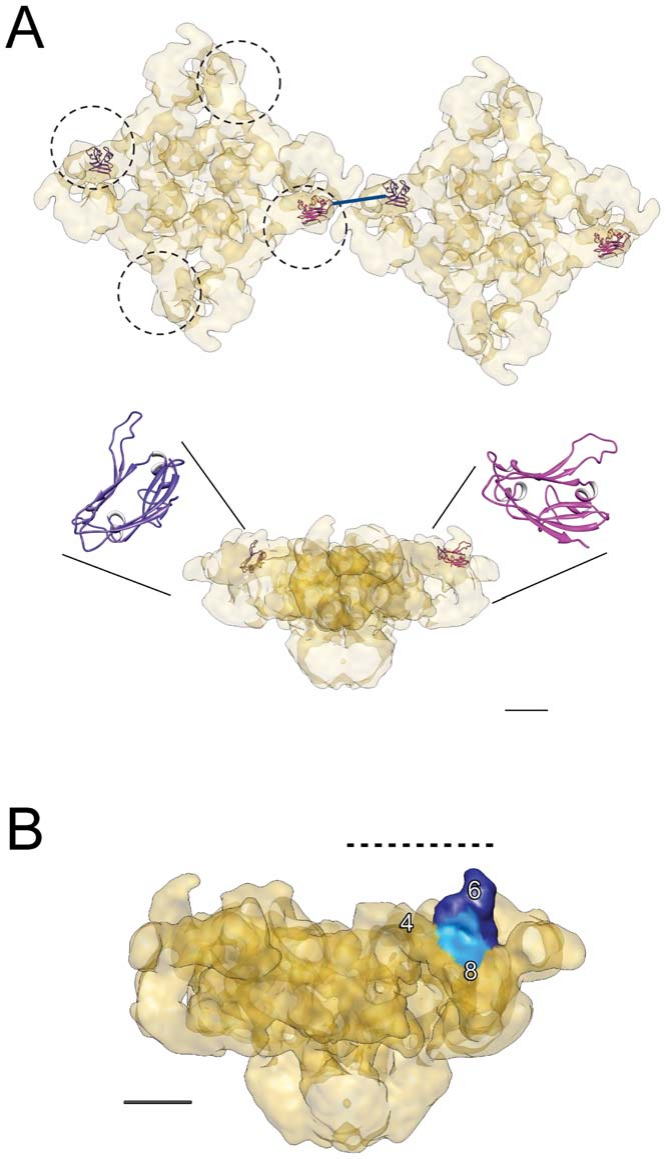
Docking of a homology model for the SPRY2 domain in the context of the RyR1-DHPR and inter-RyR1 interactions found in the triad junction. *A.* Docking of a homology model for the SPRY2 domain in the anti-SPRY2 binding site of RyR1. The two RyR1s are in the typical lattice arrangement [8] and the dashed-line circles indicate the region of overlap with the DHPR [36]. The two SPRY2 orientations within the RyR1 originate from two slightly different starting locations for the antibody-binding site: that obtained from the unsymmetrized 3D volume (purple) and that obtained from the fourfold symmetrized 3D volume (magenta). The RyR1 with docked SPRY2 is shown in two orthogonal orientations. The distance between two proximal SPRY2 domains of two neighboring RyR1s measures 60 Å (blue line). *B.* Domains of RyR1 relevant for the interaction with the DHPR: DR2 region (domains 6–8, light blue [39]), and region of overlap with DHPR [36] delimited by dashed line. The SPRY2 domain (domain 6) is shown in dark blue. The side view of RyR1 is rotated 45° around the fourfold axis with respect to the side views shown in panel A and Fig. 3, panels B–D. Scale bars, 5 nm.

For illustration purposes, we have docked the homology model of the SPRY2 domain of RyR1 [20] in the RyR1 density map, using as docking site the anti-SPRY2 binding sites resulting from this study (Fig. 4A, see Methods section for details). The SPRY2 model was initially placed in the high-intensity spots (indicated by arrows in Fig. 3E) for both the symmetry-free and fourfold symmetry reconstructions (in the vicinity of domain 6, according to Figure 3E) with three initial different orthogonal orientations (90°, 0°, −90°) of the loop 2 in respect to the RyR1 reconstruction in order to avoid favoring any specific orientation of the loop. Thus, we had six initial positions. The system was allowed to iterate until reaching a solution. Six different solutions were obtained (data not shown). Five of these solutions docked in domain 6. The other solution docked in domain 4 (adjacent to domain 6). Four out of six solutions displayed the SPRY2 loop towards the cytosolic side. These solutions with an exposed SPRY2 loop are the only ones that can explain the binding of the anti-SPRY2-A antibody, specifically designed against this loop, in the folded RyR1. From the different dockings that we obtained, we have subjectively displayed two of the docking solutions with the SPRY2 loop oriented towards the cytosol (Fig. 4A).

## Discussion

### Recovering the Signal when a Symmetric Protein is Partially Decorated

In this study, we have mapped the SPRY2 domain of RyR1 (residues 1085–1208) in the vicinity of domain 6 by antibody labeling in combination with single-particle cryo-EM. This is the first 3D localization of a SPRY2 domain in any known RyR isoform. The 3D reconstruction scheme had to be modified to take into account the signal “dilution” caused by partial decoration of equivalent, symmetrically located binding sites. For this, the extra mass corresponding to the antibody was computationally “moved” to a specified repeat of the structure as follows. Since for a symmetric shape there are several redundant configurations (in our case four) of the Euler angles that yield the same 2D projection, for every decorated raw image where the antibody was visible and did not fall in a specified corner of the RyR1, we replaced its Euler angles for the symmetrically related Euler angles that would place the antibody on the specified corner. If the raw image had more than one antibody bound, this was repeated by providing the symmetrically related Euler angles that would place this second bound antibody in the specified location, and so on. Finally, the 3D reconstruction was performed with all antibodies back-projected to the specified repeat.

The above method works as long as the antibody is visible and always binds to the same site. To establish this we first made a rough identification of the 3D position of the antibody relative to the RyR1 by creating a simulated 3D of RyR1 with four antibody-sized spheres located at four equivalent positions and projecting this simulated model in all orientations. Then once the RyR1 in the raw image was matched to the corresponding projection, the position of the projected spheres was compared to the position of the extra mass (antibody). The four spheres were then placed in other locations and the process was repeated until the match between the set of simulated projections and the set of raw images was optimal (Figure S1), which indicated the approximate 3D location of the antibody. With this new method it has been possible not only to identify the antibody in the individual particles but it has also been possible to recover the signal in the context of the 3D structure.

### Location of the SPRY2 Domain in the Context of the 3D Structure of the RyR1

SPRY domains have been shown to mediate protein-protein interaction processes involved in diverse cellular functions [11], [12], [13], [14], [15], [16]. The SPRY domains of several proteins have been recently crystallized, revealing a high structural conservation for this domain. A distinctive feature of SPRY domains for the protein-protein interaction specificity is the presence of an unstructured and flexible loop between β-strands (Fig. 4A). The flexible loop is thought to mediate specific protein-protein interactions. This loop shows conformational exchange in intermediate time scales, which appears to be important for protein-protein interactions [35].

The 3D location of SPRY2 within domain 6 of RyR1 found in this study, near the corners of the square prism-shaped cytoplasmic domain and oriented towards the T-tubule, overlaps with regions that contribute to the interaction between the DHPR and RyR1 identified in several previous studies (see Fig. 4B). First, the proposed positioning of the DHPR tetrads with respect to the RyR1 found by TEM thin section and freeze-fracture studies [8], [36] locate these in domains 5-6-9. Second, studies using green fluorescent protein tags inserted along the RyR1 sequence have shown that the divergent region 2 of RyR1 (DR2, residues 1342–1403), a sequence crucial for the RyR1-DHPR coupling [37], [38], was located between domains 6 and 8 of RyR1 [39]. The TEM thin-section studies also indicate that RyR1s form a two-dimensional lattice [8]. In the lattice, two SPRY2 domains from two adjacent RyR1s are facing each other (Fig. 4A). However it is unlikely that SPRY2 domains have a direct role in these inter-RyR1 interactions because two neighboring SPRY2 domains are located at a relatively large distance of each other, around 60 Å. Finally, the location of the SPRY2 domain on the peripheral region of the cytoplasmic domain makes it also widely accessible to other protein binding partners in the cytoplasm.

In conclusion, our methodological approach has allowed us to extract detailed and relevant information out of very limited amount of data. Our antibody labeling and 3D mapping of RyR1 provides new and deeper insight on the location of the SPRY2 domain in the RyR1 structure and in the context of the RyR1-DHPR interaction. Until the structure of big proteins such as RyR1 can be resolved by higher resolution techniques such as X-ray crystallography, cryo-EM appears to be the best feasible alternative to identify regions in the 3D structure of proteins.

## Materials and Methods

### Source for the Antibodies against the SPRY2 Domain

Anti-SPRY2-A: A peptide encoding a SPRY2 domain loop between residues Arg^1106^ to Leu^1120^ was synthesized and coupled to Keyhole Limpet Hemocyanin by the Australian Cancer Research Foundation Biomolecular Resource Facility at the John Curtin School of Medical Research. The SPRY2 loop peptide was used as an immunogen for the preparation of anti-SPRY2-A polyclonal antibodies. Rabbit polyclonal antiserum was generated by standard techniques after three weekly injections of the antigen emulsified in Freund's adjuvant. The specific anti-SPRY2 antibodies were purified from the crude antiserum by affinity purification on SPRY2 immobilized on nitrocellulose as described previously [40].

Anti-SPRY2-B: Recombinant SPRY2 domain protein for use as an antigen for rabbit antiserum production was expressed in *E.coli*. A cDNA fragment encoding residues from Phe^1075^ to Ser^1210^ was amplified from the *Oryctolagus cuniculus* (European rabbit) RyR1 cDNA [41]. The amplified cDNA was cloned between the *EcoR1* and *Sal1* sites of the expression vector pHUE [42]. To facilitate cloning, additional bases were included at the 5′ and 3′ ends of the cDNA that resulted in the inclusion of non-native Ser and Glu residues at the N-terminal of the finally purified protein. The use of the pHUE vector and the purification of expressed proteins have been described in detail [42]. Briefly the recombinant protein was initially expressed as a polyHis-ubiquitin-SPRY2 fusion protein that was purified by affinity chromatography on Ni-iminodiacetate agarose. Subsequently the N-terminal polyHis-ubiquitin tag was removed by cleavage with the catalytic core of the ubiquitin cleaving enzyme Usp2. Finally, the polyHis–ubiquitin tag and the Usp2 fragment were removed by a second round of Ni agarose chromatography. The purified SPRY2 protein was used for the preparation of polyclonal (anti-SPRY2-B) and monoclonal antiserum (anti-SPRY2-C).

Anti-SPRY2-C: Monoclonal antibodies were developed in the laboratory of Professor Yoshinobu Eishi by a standard protocol [43] after immunization of BALBc mice with purified recombinant SPRY2 protein. Hybridoma cell lines producing anti-SPRY2 antibodies were checked by enzyme-linked immunosorbent assay (ELISA) with the recombinant SPRY2 protein. Hybridomas giving positive results were screened by immunohistochemistry with formalin-fixed and paraffin-embedded tissue sections of skeletal muscle.

### RyR1 Purification

RyR1 from rabbit skeletal muscle was purified as described previously [21], [22]. Briefly, the solubilized RyR was purified from SR vesicles using gel filtration and sucrose gradient, followed by concentration on a heparin column. This preparation yielded a single band compatible with a MW of 565 kDa.

### Preparation of RyR1-anti SPRY Complexes for cryo-EM

Antibodies and RyR1 were mixed at a molar ratio of 10 (antibody:RyR1 tetramer) and incubated at 4°C for a time period ranging between 3–9 hours. The final RyR1 concentration was of 2.26 mg/ml in all cases. The final buffer was 20 mM MOPS pH 7.4, 0.15 M NaCl, 0.5% CHAPS, 2 mM EGTA, 2 mM DTT, conditions known to favor the RyR1 in the closed conformation [21], [22], and a protease inhibitor cocktail.

### Immunodetection

Rabbit muscle vesicles containing native RyR1 were transferred to PVDF membranes by means of vacuum (dot-blot). Purified RyR1 from rabbit muscle vesicles was resolved by means of SDS-PAGE and electrotransfered to PVDF membranes in a denatured state (Western blot). RyR1 was immunodetected in both native and denatured states by incubation of PVDF membranes with rabbit raised anti-SPRY2-A and anti-SPRY2-B, and with mouse raised anti-SPRY2-C and 34C (abcam ab2868) antibodies. The detection was carried out using both anti-rabbit and anti-mouse secondary antibodies and a horseradish peroxidase-based chemiluminiscent detection kit (Supersignal West Dura, Thermo Scientific).

### Cryo-EM and Single-Particle Image Processing

A 5 µl aliquot of the RyR1-antibody incubation mixture was adsorbed to a holey grid (either Protochips ™ or quantifoil™), and the excess of buffer blotted off with Whatman 540 filter paper, blotting time 4″. The samples were vitrified by plunging the grid into liquid ethane using either an FEI Vitrobot™ device, with the main chamber at 80% humidity. Cryo-electron microscopy was performed on a FEI Tecnai F20 operated at 200 kV under low dose conditions and a magnification of 50,000×. Defocus range varied between 2.5 and 4 µm. A total of 11, 35 and 49 micrographs for anti-SPRY2-A, anti-SPRY2-B, and anti-SPRY2-C, respectively, were recorded on Kodak SO-163 film. Micrographs were digitized in a Zeiss SCAI scanner at a step size of 7 µm or 1.4 Å per pixel, and subsequently binned down to a pixel size of 2.8 Å. A total number of 189, 490 and 1331 particles for anti-SPRY2-A, anti-SPRY2-B, and anti-SPRY2-C, respectively, were visually selected and windowed with BOXER [32]. All subsequent image processing steps were carried out using SPIDER [33]. In brief, particles with any anti-SPRY2 antibody bound were visually inspected yielding a dataset formed by 90 particles. After ensuring that the three antibodies bound to the same region of RyR1, these particles were matched to a reference with defined Euler angles obtained from the projection of a low-resolution reference 3D volume of RyR1. The Euler angles for these particles were re-computed in order to place the antibody-binding site in a single repeat of the (fourfold symmetric) RyR1, yielding a total of 124 particles. These particles were used to determine a non-symmetric and a fourfold symmetric (assuming RyR1's fourfold symmetry) 3D reconstruction for the antibody-bound particles. In the density map, the steep region in the density gradient marks the boundaries of the macromolecule. We choose the mid point (threshold of 0.25 in our case) as the density threshold for the isosurface representations. The 3D reconstructions were low-pass filtered to 30 Å resolution using a Fermi filter.

### Visualization and Docking

Chimera [44] was used to display the 3D volumes and atomic models in all figures. For visualization purposes and to show more accurately the distinctive features of RyR1, a 3D map of RyR1 at 10.3 Å resolution is displayed in Figures 3B, 3C, 3E and 4. The homology modeled SPRY2 domain [20] was docked in the RyR1 cryo-EM map using the correlation method in “Fit in Map” tool from Chimera [45]. A 10.3 Å resolution map of the SPRY2 model was generated and was allowed to freely rotate and move using the full density range. The correlation of the fit was used as the weighing function and the system was allowed to iterate until it stabilized.

## Supporting Information

Figure S1 **Complete set of identified RyR1 single-particles incubated with anti-SPRY2 antibodies presenting additional mass (indicated by white arrows).** Unprocessed RyR1 particles incubated with the specified anti-SPRY2 antibody (anti-SPRY2-A, anti-SPRY2-B, anti-SPRY2-C; upper row), and calculated 2D projections (bottom row) for RyR1 containing four 30 Å radius spheres at the preliminary proposed binding region for the anti-SPRY2 antibody.
(PDF)
