# Supplementary figures and images for: 3D Mapping of the SPRY2 Domain of Ryanodine Receptor 1 by Single-Particle Cryo-EM
Source: PLoS One. 2011 Oct 5;6(10):e25813. doi: 10.1371/journal.pone.0025813 (PMC3187800; doi:10.1371/journal.pone.0025813)

$\alpha$ -SPRY2-A

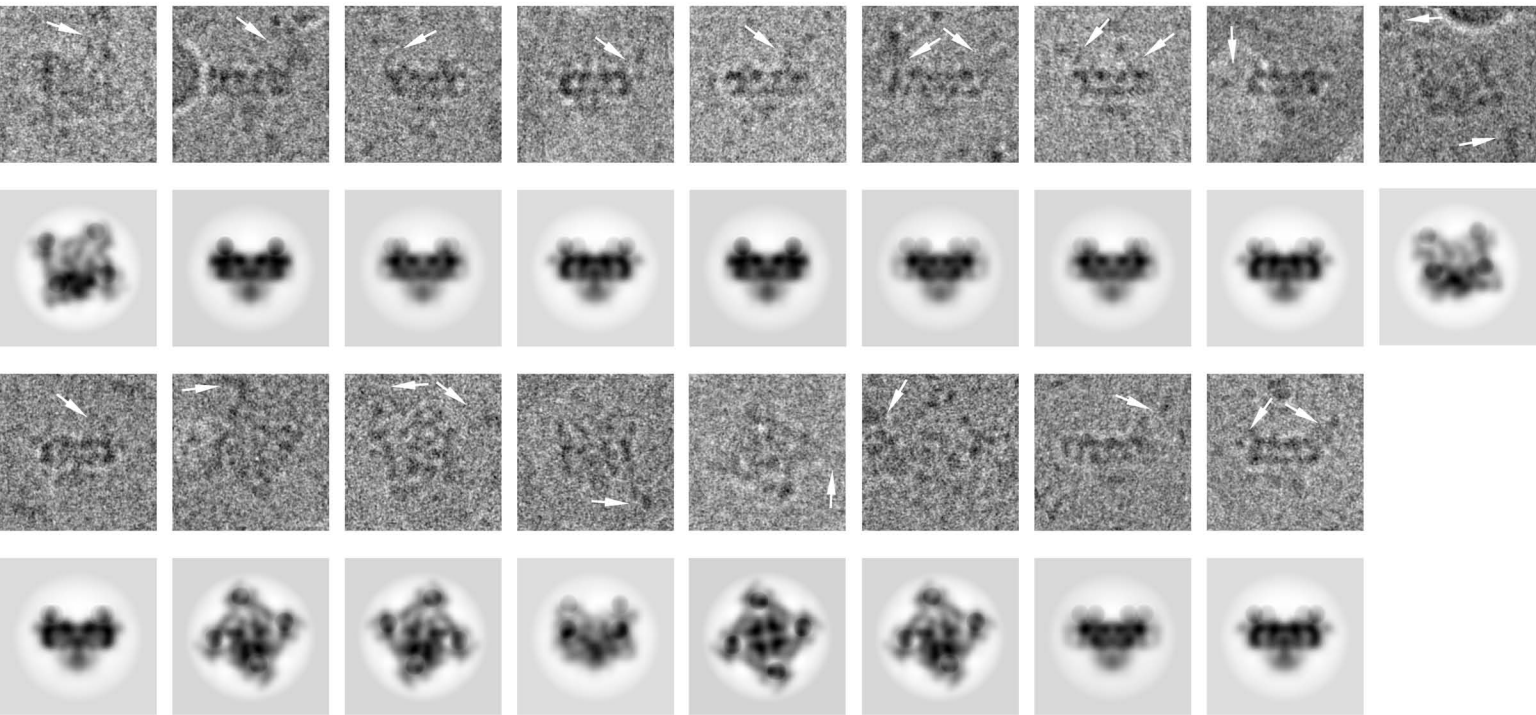

$\alpha$ -SPRY2-B

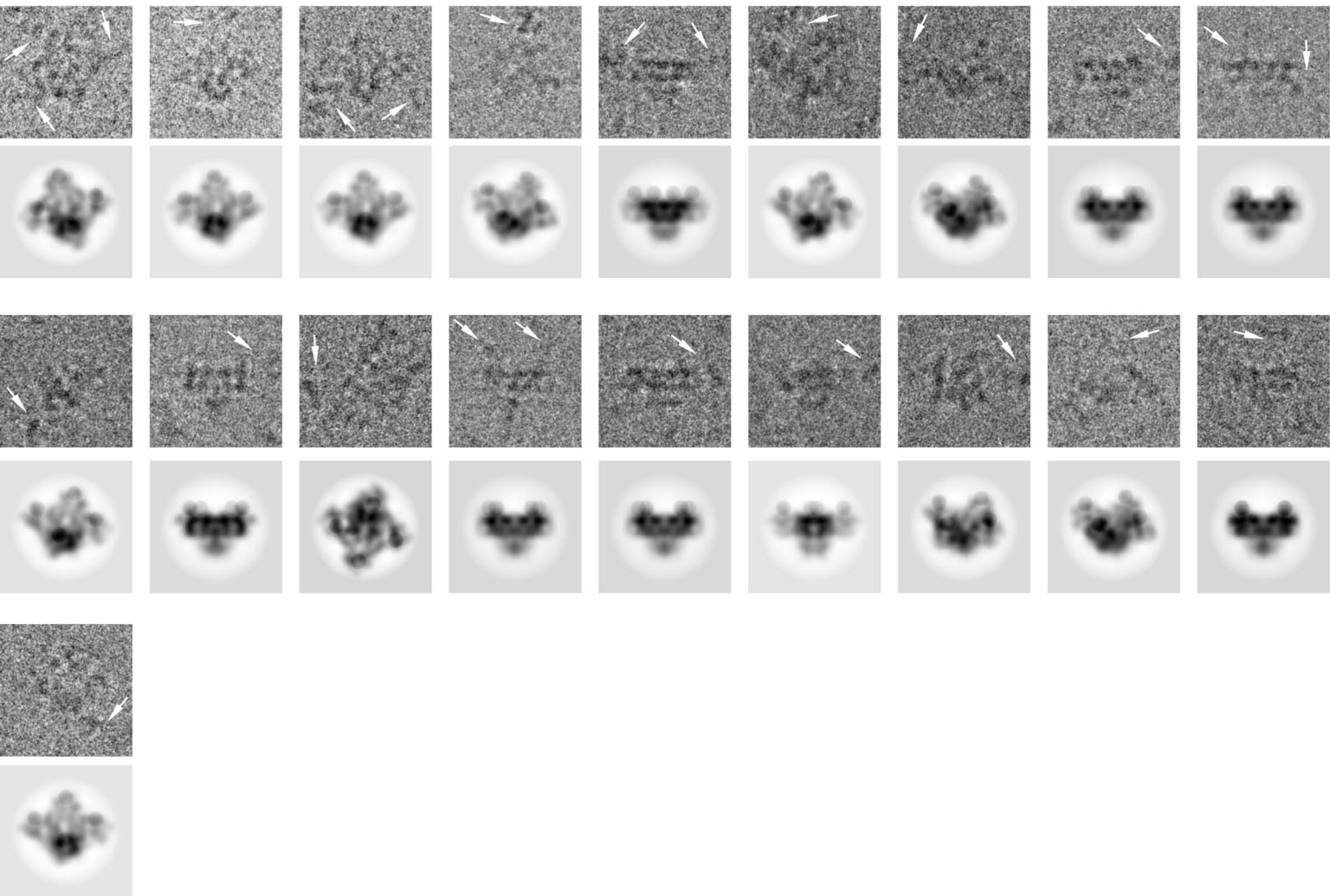

$\alpha$ -SPRY2-C (I)

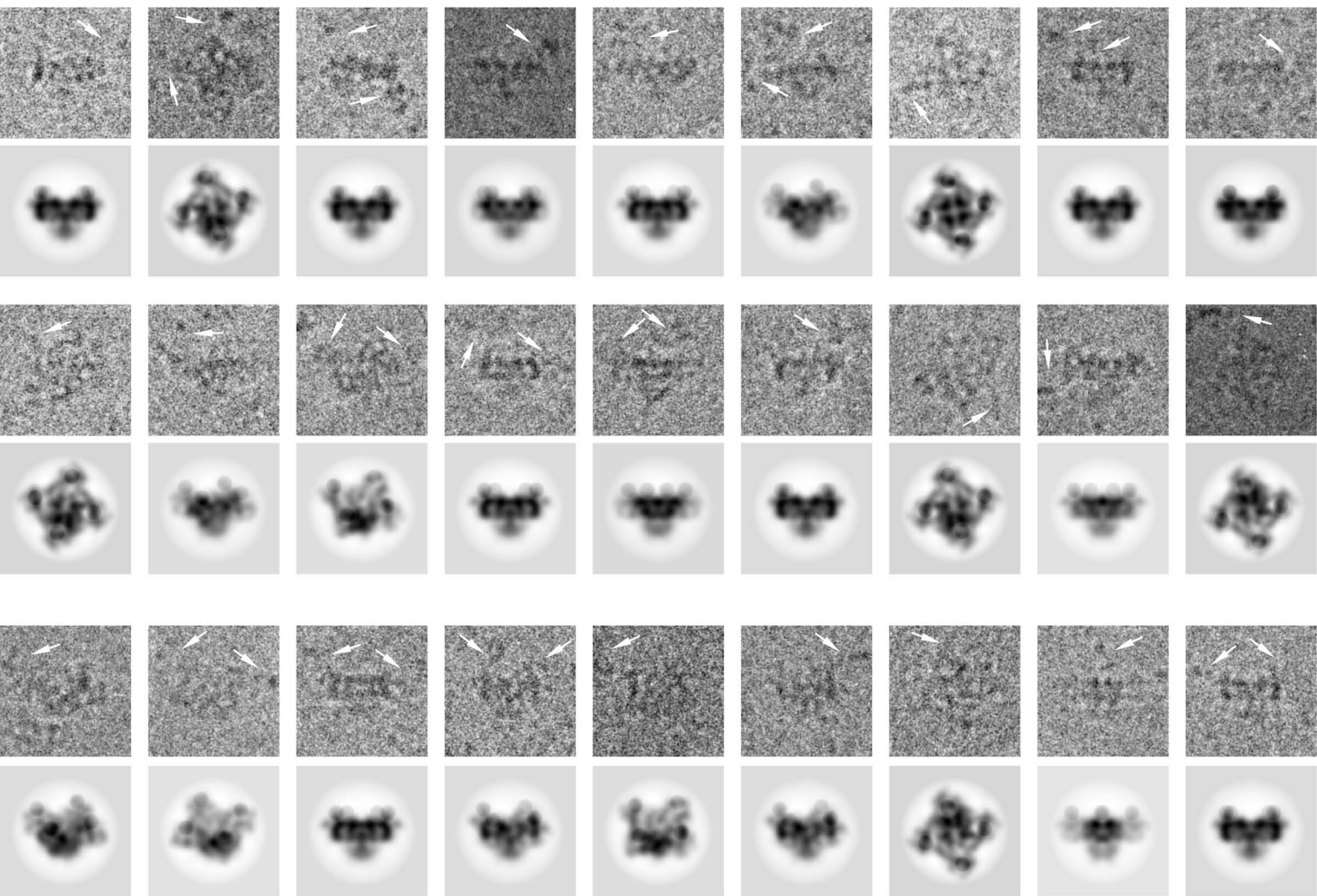

$\alpha$ -SPRY2-C (II)

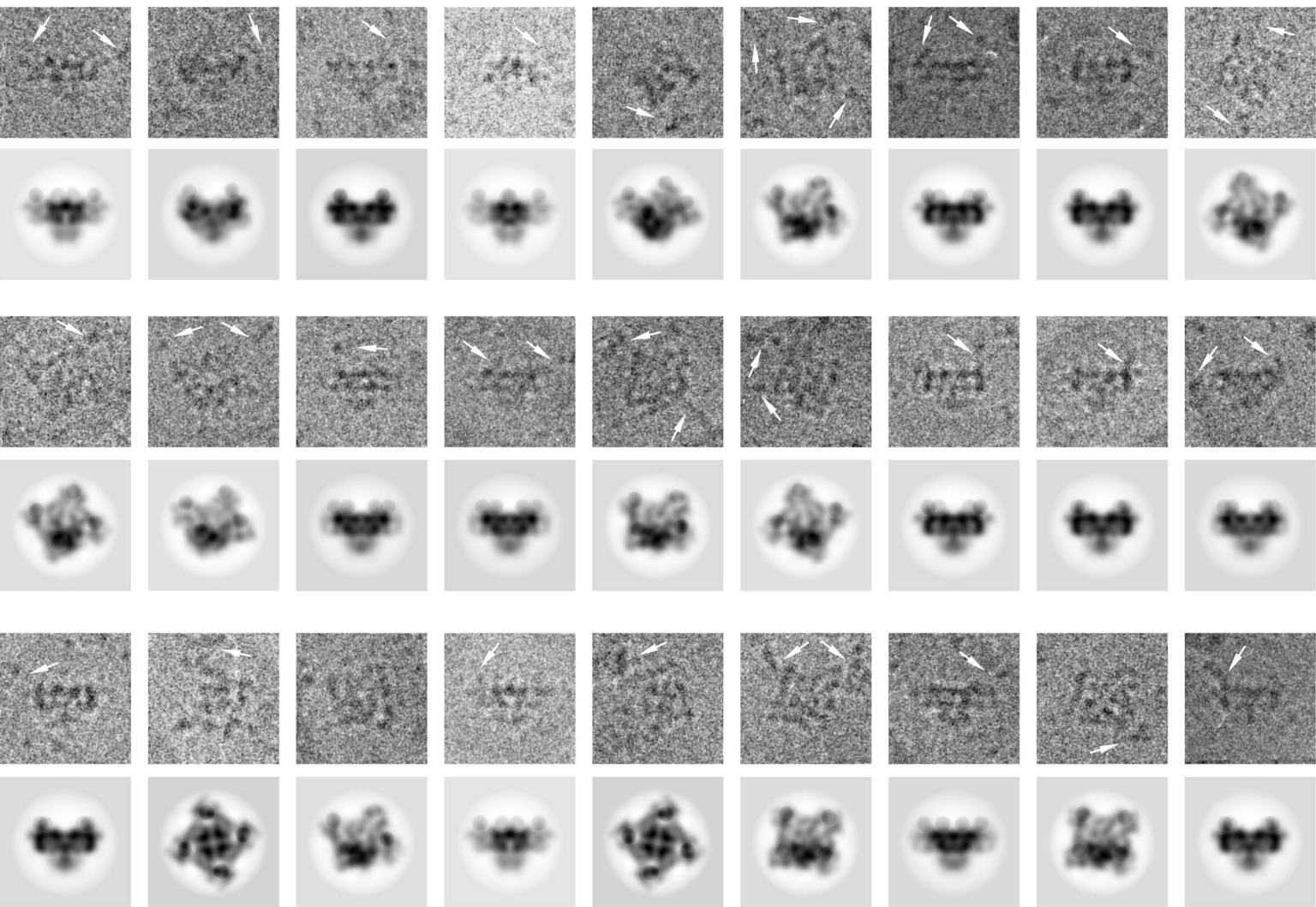

Supplement: Figure S1 — Complete set of identified RyR1 single-particles incubated with anti-SPRY2 antibodies presenting additional mass (indicated by white arrows). Unprocessed RyR1 particles incubated with the specified anti-SPRY2 antibody (anti-SPRY2-A, anti-SPRY2-B, anti-SPRY2-C; upper row), and calculated 2D projections (bottom row) for RyR1 containing four 30 Å radius spheres at the preliminary proposed binding region for the anti-SPRY2 antibody. (PDF) [file pone.0025813.s001.pdf]
